# The Effect of Flexible Pavement Mechanics on the Accuracy of Axle Load Sensors in Vehicle Weigh-in-Motion Systems

**DOI:** 10.3390/s17092053

**Published:** 2017-09-07

**Authors:** Piotr Burnos, Dawid Rys

**Affiliations:** 1 Department of Measurement and Electronics, AGH University of Science and Technology, 30-059 Krakow, Poland; 2 Faculty of Civil and Environmental Engineering, Department of Highway and Transportation Engineering, Gdansk University of Technology, 80-263 Gdansk, Poland; dawrys@pg.gda.pl

**Keywords:** Weigh-in-Motion, axle load sensors, pavement response, overloaded vehicles, measuring of axle loads

## Abstract

Weigh-in-Motion systems are tools to prevent road pavements from the adverse phenomena of vehicle overloading. However, the effectiveness of these systems can be significantly increased by improving weighing accuracy, which is now insufficient for direct enforcement of overloaded vehicles. Field tests show that the accuracy of Weigh-in-Motion axle load sensors installed in the flexible (asphalt) pavements depends on pavement temperature and vehicle speeds. Although this is a known phenomenon, it has not been explained yet. The aim of our study is to fill this gap in the knowledge. The explanation of this phenomena which is presented in the paper is based on pavement/sensors mechanics and the application of the multilayer elastic half-space theory. We show that differences in the distribution of vertical and horizontal stresses in the pavement structure are the cause of vehicle weight measurement errors. These studies are important in terms of Weigh-in-Motion systems for direct enforcement and will help to improve the weighing results accuracy.

## 1. Introduction

### 1.1. Background

**Figure 1:**
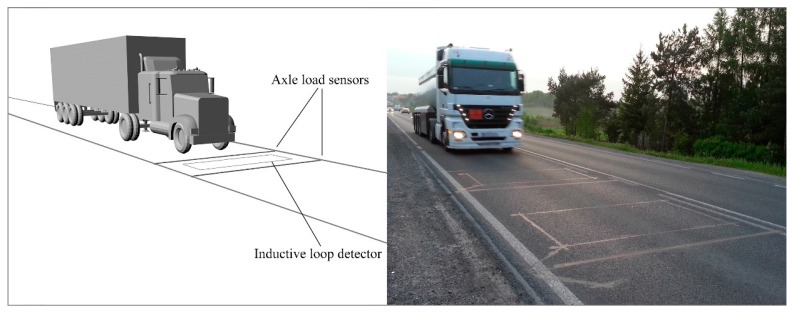
Diagram and photo of a WIM site.

Research proved that the detrimental effect of one heavy vehicle on the pavement structure is equivalent to the same effect caused by tens of thousands of passenger cars. One overloaded heavy vehicle causes several times greater fatigue damage to the pavement structure than a properly loaded one. This statement results from the fact that the damaging effect increases with approximately the fourth power of the axle load. The fundamental question is: what can we do to protect the road from increased distress caused by overloaded vehicles? In order to protect investments in roads and bridges against premature failure caused by vehicle overloading, numerous steps have been taken. Vehicles’ static gross weight and axle load enforcement is one of the steps widely used around the world. Static weighing of vehicles is characterized by a high accuracy with a relative error of 1%, but the effectiveness of such controls is small. Authorities need at least one hour to control one vehicle at the roadside and during one working day just six vehicles can be controlled. An alternative approach to weight control of vehicles is to use the Weigh-in-Motion system (WIM). Axle load sensors of such systems are embedded directly in the pavement of the road, perpendicular to the direction of the traffic flow (see Figure 1). Such construction of the WIM systems allows for the weighing of vehicles in motion without the need to stop them, and makes the control effective because each vehicle passing over the WIM site is weighed. This is the advantage of the WIM system as compared to static scales, and the lack of additional constraints on vehicle speed is considered to be the most attractive feature. Unfortunately this is achieved at the expense of the low accuracy of weighing results, which is the drawback of WIM systems. Our experience shows that the relative error reaches as much as 15% for axle load and 5% for gross vehicle weight (GVW). This is the reason why WIM systems are not currently in use for direct enforcement of overloaded vehicles. Another problem is that there are no relevant legal regulations allowing such application of WIM systems. One of our papers is dedicated to this problem [1]. However, WIM systems are used for preselection and identification of potentially overloaded vehicles to direct them to control on certified, static scales. Figure 1 shows a diagram of a WIM system and a photo of a Polish WIM site.

**Figure 2:**
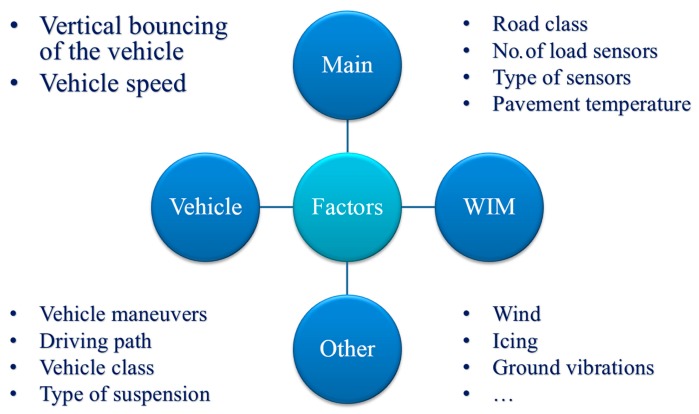
Factors affecting the weighing accuracy with division according to the source of their occurrence (vehicle or WIM system) or degree of influence (main or other).

In the initial period of the development of WIM systems, the variable wheel load of a moving vehicle was considered to be the only cause of the serious inaccuracy of weighing results. However, a more insightful study demonstrated that there were additional factors interfering with the measurement [2] including: road roughness, temperature of the pavement, value of wheel load, vehicle speed and weather conditions, etc. These factors affect all kinds of axle load sensors, regardless of the sensor technology. We divided them according to the source of their occurrence (vehicle or WIM system) or degree of influence (main or other). Figure 2 shows such a division.

**Figure 3:**
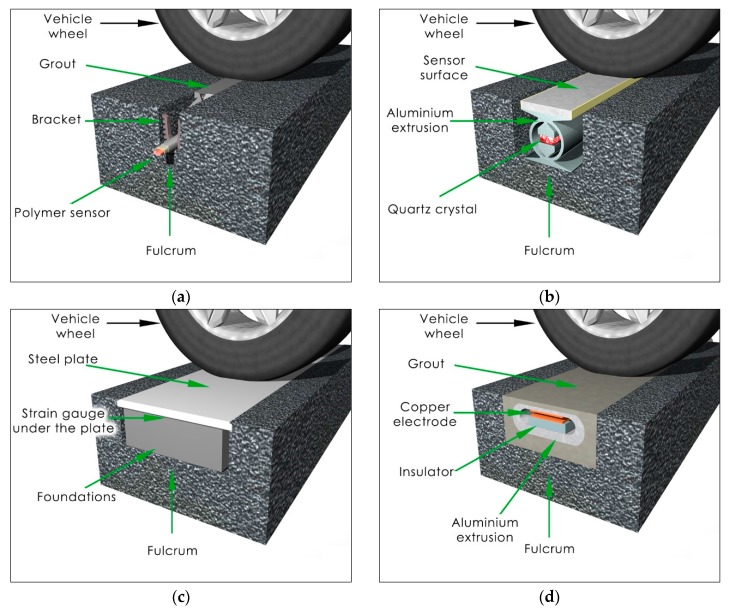
(**a**) Polymer sensor installed below the pavement surface; (**b**) Quartz sensor installed on the level of the pavement surface; (**c**) Bending plate sensor installed on the level of the pavement surface; (**d**) Capacitive sensor installed on the level of the pavement surface.

Axle load sensors are embedded in the pavement perpendicular to the traffic flow direction. Nowadays in WIM systems, sensors made by four different technologies are used: piezoelectric (polymer or ceramic), quartz, bending plate and capacitive [3]. One promising technology is fiber-optic, and despite the fact that such sensors are not in use yet, information about their tests can be found in the literature [4]. From the point of view of the installation method, WIM sensors are divided into two groups:

- Sensors installed in a small cut in the pavement at a depth of 2 to 10 cm depending on the sensor type (Figure 3a). In this case, the sensor does not have direct contact with the vehicle wheel and the axle load is transmitted to the sensor by the pavement and installation grout (which is used to fill up the cut). Polymer and piezo-ceramic sensors are mounted in this technology.
- Sensors installed in a cut in the pavement on the level of the pavement surface (Figure 3b–d). In this case, the sensor has direct contact with the vehicle wheel. Quartz, bending plate and capacitive sensors are mounted in this technology.

Regardless of the mounting method, the fulcrum for the sensor is the pavement. In the case when the sensor is installed under the road surface, the wheel load is transmitted to the sensor by the pavement and installation grout. Both facts justify our hypothesis that the pavement plays an important role in the measuring process. This is an unusual situation because the pavement, which is a part of the road, becomes a part of the weighing system. In this way, the pavement properties affect the properties of the whole WIM system.

Taking into account the above considerations, we propose splitting the weighing errors into two groups:

- Sensor intrinsic error—related to the change in the sensor’s electrical parameters under the impact of temperature change.
- Pavement/sensor complex external error—which is a combination of the sensor intrinsic error and additional errors which occur after the sensor installation in the pavement. The source of these errors is the pavement, as its properties depend on the temperature and duration of force applied by the vehicle wheel on the pavement/sensor complex.

The problem with the accurate weighing of vehicles in motion ensues from the high value of the pavement/sensor complex external error. We have written about this in our article [5]. Preliminary research shows that three factors have a significant influence on the pavement/axle load sensor complex and thus the weighing result accuracy:

- Changes in pavement temperature.
- Duration of forces applied to the pavement/axle load sensor complex, dependent, in turn, on the speed of the vehicle being weighed.
- Stress on the tyre-pavement contact area, which depends on wheel force values.

### 1.2. Objective and Scope

The phenomena which occur at the junction of the pavement/sensor complex have not yet been examined in detail. The aim of our study is to fill this gap in the knowledge. These studies are important in terms of Weigh-in-Motion (WIM) systems for direct enforcement and will help to improve the accuracy of the weighing results. The analysis is focused on flexible (asphalt) pavements due to the fact that most WIM systems are mounted in such pavements.

This paper is divided into two parts. The first part concerns the identification of the problem on the basis of field observations. The field observations are based on the analysis of data from in service Weigh-in-Motion systems and the identification of relative error caused by changes of temperature and vehicle speed. The second part of our paper is the theoretical explanation of the observed phenomena with the objective of identifying the source of the error observed in the field. The explanation is based on the analysis of a mechanistic model of the flexible pavement structure and the behaviour of asphalt mixes under different load conditions.

## 2. Literature Review

The improvement of WIM system accuracy is a move towards the WIM system certification by the National Metrological Institutes. This will allow WIM systems to be used for the direct enforcement of weight restrictions which will lead to a reduction in the number of overloaded vehicles [6,7,8,9,10]. The reduction of overloading contributes to a reduction in pavement maintenance costs by extension of the pavement serviceability period [11,12,13,14,15]. According to studies [11,14,16] based on data analysis from 12 Polish WIM stations, the average percentage of overloaded vehicles in heavy traffic is 15% to 30% and these vehicles are responsible for up to 70% of the fatigue damage of pavement structures. Studies carried out by Taylor et al. [15] in New York State (USA) showed that the percentage of overloaded vehicles was reduced from 30% to 2% after the enforcement level was increased. This means that the pavement fatigue life could be approximately doubled, bringing economic and social benefits. This proves the importance and necessity of improving WIM system accuracy.

Weigh-in-Motion systems are also able to provide statistical knowledge of the traffic that may be used for traffic management, traffic flow optimization and the design of road infrastructure for pavements [11,14,17,18] and bridges [19].

Regarding the influence of temperature on the weighing results, a few studies have been performed in the past. In 2001 Papagiannakis, Johnston and Alavi published results [20,21] of the laboratory and field evaluation of the fatigue characteristics of piezoelectric axle load sensors. In these experiments, the sensitivity of the sensors’ output signal to the pavement temperature was investigated. The studies showed a relationship between the signal level and the pavement temperature, but no explanations of these phenomena were given.

The temperature sensitivity of axle load sensors is also addressed in [22]. The paper describes laboratory and field tests of polymer WIM sensors. Experiments proved a relationship between the sensors’ output signal and the pavement temperature. The signal level increased with the increased temperature of the asphalt concrete pavement. The authors evaluated the temperature coefficient of this relationship as equal to 0.162 volts/degree.

The polymer sensors’ sensitivity to the pavement temperature changes and vehicle speed changes is also described in paper [23]. The results presented showed that polymer and ceramic WIM sensors are temperature sensitive, while quartz sensors are insensitive to temperature. Regarding the influence of vehicle speed, the authors concluded that the polymer sensors were the least sensitive to speed change, the quartz sensors had better accuracy at higher speeds and the results for the ceramic sensors were more scattered than for the other two types of sensors overall.

Similar research was conducted by Vaziri [24,25]. The aim of that research was to test piezoelectric axle load sensors under different environmental conditions and vehicle behaviour during weighing. Vaziri with his team conducted field tests on two sites equipped with quartz and piezoelectric (polymer and ceramic) sensors. The air temperature was measured instead of the pavement temperature, thus, the reliability of the presented results was limited. Regarding the effect of the vehicle speed on the sensors’ output signal, tests were conducted for three speeds: 30, 50 and 70 km/h.

The properties of WIM sensors embedded in the pavement were also examined by one of the authors of this paper. A team from AGH University of Science and Technology (AGH-UST)—during the last ten years—conducted a series of tests at a site equipped with 16 polymer sensors and in the laboratory [26]. Long-term tests clearly showed a strong relationship between the pavement temperature and weighing results. A model of this relationship was first introduced by Burnos in 2008 [27] and further investigated in [28]. In the latest paper [5], Burnos and Gajda presented a detailed analysis of the thermal properties of axle load sensors mounted in the pavement. We conclude that the pavement temperature is a significant influencing factor of weighing result accuracy and this phenomenon concerns all examined sensors: polymer, quartz and bending plates.

None of the above-mentioned papers explains the phenomenon of sensor sensitivity to pavement temperature and vehicle speed changes. Some attempts were made in research conducted by IFSTTAR and described in [29]. The authors proposed a numerical model that represents the axle load sensor electric response. However, the phenomena which occur in the pavement/axle load sensor complex, under the impact of changes in pavement temperature or vehicle speed, were not considered. In another paper [30], the authors described the results of the evaluation of different WIM sensors. Their results confirmed the sensors’ output signal dependency on the temperature and vehicle speed.

The aim of our study is to fill the gap in the knowledge and to explain the phenomenon of the sensors’ signal dependence on the pavement temperature and vehicle speed. This will help to counteract the adverse effects and improve the accuracy of vehicle weighing in WIM systems.

## 3. Field Tests

As we mentioned before, the problem with the accurate weighing of vehicles in motion ensues from the high sensitivity of the WIM systems to pavement temperature changes and vehicle speed. To assess the impact of these factors on the weighing results, we analysed data from three roadside WIM systems located in Poland:

- Site No. 1 on road 81 near the city of Gardawice—equipped with 16 lines of Roadtrax^®^ Brass Linguini^®^ polymer load sensors made by TE Connectivity Ltd [31].
- Site No. 2 on road 79 in the city of Rudawa—equipped with 2 lines of Lineas^®^ quartz sensors made by Kistler [32].
- Site No. 3 on road 6 in the city of Zabowo—equipped with 2 lines of PAT DAW 100^®^ bending plate sensors made by International Road Dynamics^®.^

At each site, the data were collected for at least 6 months. For the accuracy assessment of each WIM system, we used the reference vehicles method [27]. This is a statistical method based on the assumption that the first axle load in a given class of vehicles has low random variability, constant mean value and is most weakly correlated with loads of other axles and gross vehicle weight (GVW). This means that the change of the first axle load from vehicle to vehicle is small, regardless of the carried load. As the random variability is small, the loads exerted by the first axle of these vehicles are treated as reference values for system accuracy assessment. In Poland, five-axle articulated vehicles, including two-axle tractors and three-axle semi-trailers, are categorized into this class.

As a quantitative assessment of the accuracy, we used the relative error of weighing results *δ* which is computed from Equation (1):

(1) $$\delta =\frac{1}{N}\sum_{i=1}^{N}\frac{Ldyn_{i}-Lref}{Lref},$$

where:

- *Ldyn*_i_—first axle weighing result of the reference vehicle obtained from the WIM system,
- *Lref*—reference value of the axle load, in this case the mean value of the first axle load of the reference vehicles (61.670 N),
- *i* = 1, 2, …, *N*—number of weighing results of the first axle of the reference vehicles, taken at the specific value of the temperature and the vehicle speed.

**Figure 4:**
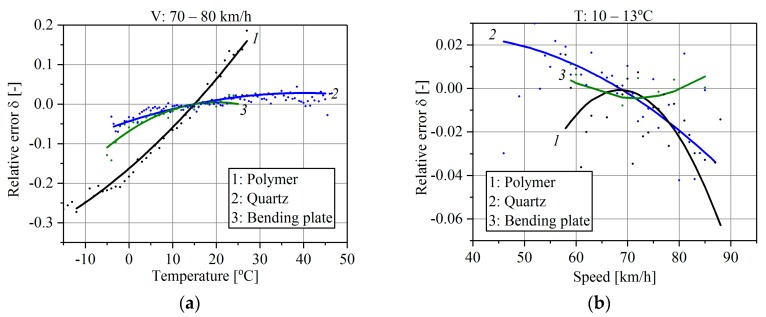
(**a**) Temperature characteristics; (**b**) Speed characteristics of polymer, quartz and bending plate sensors installed in the pavement.

The influence of temperature and vehicle speed on the weighing results in all three systems is shown in Figure 4. The characteristics illustrate the relative weighing error (for GVW or axle load) dependence on the temperature and vehicle speed. The solid lines in Figure 4 are second-order polynomial approximations (the best fit) of the relative error (1). Each approximated point is the average value of the results of weighing at least several dozen vehicles.

To distinguish temperature effects from the influence of vehicle speed on the weighing error, temperature characteristics were determined for the range of vehicles’ speeds of 70 km/h–80 km/h and speed characteristics were determined for the narrow pavement temperature range of 10 °C–13 °C. The investigated systems were calibrated at the temperature 15 °C and for the vehicle speed of 70 km/h, which yields *δ* = 0 for those values.

It should be emphasized that the presented characteristics take into account the temperature and speed influence on the pavement/sensor complex external error because those quantities affect both sensor and pavement properties. From the characteristics presented in Figure 4, a few conclusions can be formulated:

- WIM systems are temperature and speed sensitive, regardless of the sensor technology. Thus, the widely prevailing opinion among users that quartz and bending plate sensors are insensitive to changes of the pavement temperature and vehicle speed must be verified.
- Polymer sensors have the worst properties and should not be used for direct enforcement purposes. For this type of sensor, a temperature change caused a change in the weighing error of approximately 50%.
- In the case of quartz and bending plates sensors, a temperature change within the range 4 °C to 40 °C produces—as an effect—a change in the weighing error of approximately 3%.
- Weighing results for the same vehicle travelling at speeds of 55 km/h and 85 km/h differed by 6% for polymer sensors, by 4% for quartz sensors and by 1% for bending plate sensors.
- WIM systems are more sensitive to pavement temperature changes than to the vehicle speed changes. Nevertheless, speed effects cannot be neglected in light of the use of WIM systems for direct enforcement purposes.

Up to now, there is no known model of the described phenomena nor any theoretical explanation of the pavement/sensor complex behaviour under various conditions. Such a model would have two advantages: cognitive and productive. In terms of cognitive values, it would help to understand and explain phenomena responsible for the system sensitivity to the temperature and vehicle speed changes. The productive outcome of such a model would be the possibility of its application for compensation of the temperature and vehicle speed influence on the accuracy of weighing. In the next paragraph, we present theoretical explanations of observed phenomena.

## 4. Theoretical Explanation of Observed Phenomena

The wheels of a moving vehicle induce stresses, strains and deflections in the pavement. Pavement deflection is a result of stresses and strains in each layer of the pavement structure: wearing course, binding course and asphalt base. A deflection is a non-linear function of several quantities including wheel load value, contact stress in a tyre-pavement contact area, pavement layer thickness and its mechanical properties. The stiffness modulus of asphalt layers is a function of the force duration applied to the pavement/sensor complex, which depends on the vehicle speed and temperature. Thus, pavement deflection and stress distribution inside pavement structure are also functions of speed and temperature. To explain the effect of temperature and vehicle speed on the accuracy of Weigh-in-Motion systems, the analysis is divided into the following steps and purposes:

1. Response of the flexible pavement/sensor complex under wheel load to identify the stresses effecting on the WIM sensor installed inside asphalt pavement.
2. Variability of the stiffness of asphalt layers to obtain the mechanical properties used in the pavement mechanistic model and to explain the sensitiveness of asphalt mixtures to temperature and vehicle speed.
3. Analysis of pavement deflections to explain how variability of the stiffness of asphalt layers impact on stress distributions inside the pavement structure. The shape and values of the pavement surface deflection results directly from stress distributions, and illustrate well the effect of variations of temperature and speed.
4. Analysis of vertical and horizontal stresses acting on WIM sensors to identify the scale of error of wheel load measurement caused by temperature and vehicle speed, and its comparison to field observation.

### 4.1. The Response of the Flexible Pavement/Sensor Complex under Wheel Load

**Figure 5:**
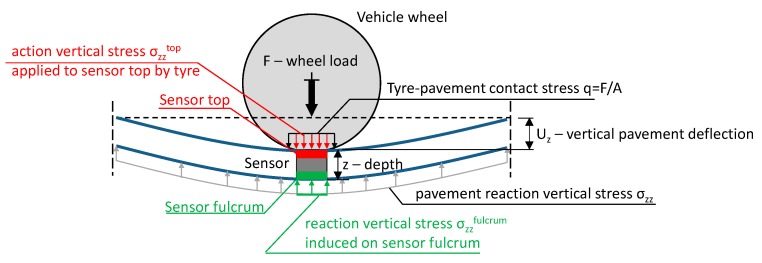
Scheme of stress effects on the WIM sensor installed inside the pavement structure.

The pavement is a part of the measuring system in WIM systems, thus the properties of the pavement affect the properties of the whole system and influence the accuracy of the weighing results. Figure 5 presents the scheme of pavement/sensor complex response under a vehicle’s wheel load for the most popular quartz sensor as an example, nevertheless the further analysis is valid for any type of sensor and installation technique. Regardless of the WIM sensor construction, the measurement signal is induced in the sensor by stresses applied on its top and fulcrum. In general, the action stress (on the sensor top) and reaction stress (on the fulcrum) are not equal due to non-uniform stress distributions in the pavement structure. Moreover, the ratio between those two stresses is not constant and depends on temperature and vehicle speed, what is proved later in the paper.

If the sensor is installed at the pavement surface level, the action vertical stress is approximately equal to the contact stress applied by the tyre. In the case of sensors mounted under the road surface, the stress value applied to the sensor top is not equal to the tyre contact stress because this stress is transmitted by the pavement and installation grout. The ratio between action and reaction stresses is not constant, which results from the stress distributions inside the pavement structure. What is more, changeable mechanical parameters of road materials, especially asphalt mixtures, cause changes in this ratio, which significantly impacts the axle load measurement error. This theoretical statement is confirmed in the field observation shown in Figure 4.

**Figure 6:**
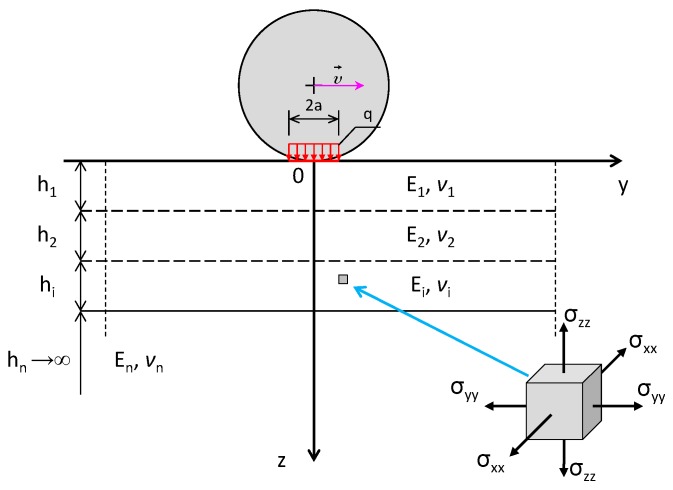
Model of the flexible asphalt pavement structure used for analysis.

The response of the pavement/sensor complex is calculated in our study with the use of the multilayer elastic half-space theory. The scheme of the pavement model is presented in Figure 6, where: *h_i_*—thickness of the asphalt layer, *E_i_*—stiffness modulus, *v_i_*—Poisson ratio, *σ*—normal stresses, *i*—number of layers, *q*—tyre-pavement contact stress, *a*—radius of a tyre footprint and *υ*—wheel speed. It is assumed that all layers of the pavement structure are homogenous, isotropic and elastic. The multilayer elastic half-space theory is described in detail in the following works [33,34] and is widely used for pavement design and analysis. The model allows stresses, strains and deflections to be calculated in each point of the pavement structure. The wheel load is applied by a circular contact area and uniform contact pressure. The shapes and values of the contact load are in accordance with the assumptions of the pavement design and analysis [17]. It is assumed that they induce the same level of stresses in the pavement as real traffic. The calculations of stresses, strains and deflections induced in the pavement by vehicle loads were performed in BISAR 3.0^®^ software developed by Shell company.

It must be noted that there are some limitations of the elastic-half space model. This solution does not allow us to model the WIM sensor installed in the asphalt layer. The mechanical properties of the sensor significantly differ from the properties of the asphalt mix. Despite the fact that the limitations of the model do not allow us to calculate the stresses directly under the base of the WIM sensor with very high precision, it has no effect on the theoretical explanation of the phenomena. For detailed analysis, the elastic half-space model should be replaced by the finitely element model (FEM). However, detailed analysis in the FEM model requires detailed input data of the mechanical properties of the WIM sensor and of the interface properties between the sensor and asphalt mix, what will be studied by the authors in future work. It also must be noted that analysis with the FEM consumes much more computational time and memory than analysis with the multilayer elastic half-space theory.

### 4.2. Variability of the Stiffness Modulus E of Asphalt Layers

**Figure 7:**
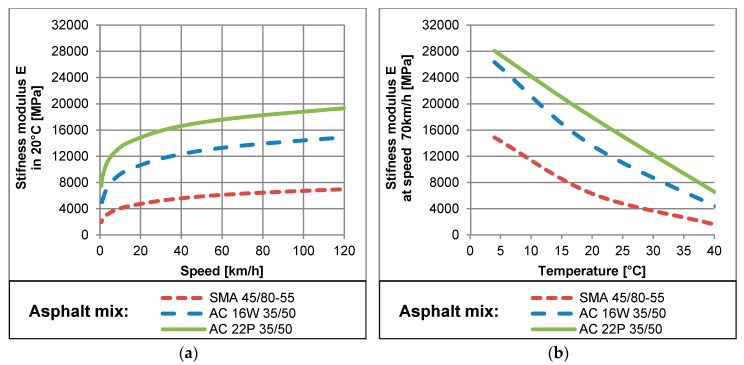
(**a**) Relationship between the stiffness modulus and vehicle speed at a constant temperature of 20 °C; (**b**) Relationship between the stiffness modulus and the temperature at a constant vehicle speed of 70 km/h, for three types of asphalt mixtures usually used in pavement structures.

The mechanical properties of the pavement structure in the elastic model are determined by the stiffness modulus *E* and Poisson ratio *v*. The stiffness modulus of asphalt layers, which includes wearing course, binding course and asphalt base, is variable. It is a function of temperature, loading time and several other factors related to the asphalt mix composition. Effects of all these factors are described in detail in publications [35,36,37,38,39]. Nevertheless, the effects of variation of the asphalt mix composition on its stiffness modulus do not impact the described phenomena and are not considered in these studies. In order to present the asphalt mix behaviour at different temperatures and under various loading times, the examples of measurements of the stiffness modulus are presented in Figure 7. The stiffness modulus values were measured in the Simple Performance Test (SPT) device according to the methodology described in [40]. The tests were performed for specimens of the asphalt mixes typically used in Poland: stone mastics asphalt (SMA) with polymer-modified bitumen 45/80-55 for wearing course and asphalt concrete AC16W and AC22P with neat bitumen 35/50 for binding course and asphalt base, respectively. It should be noted that for other samples, mixes or test schemes, the values of results would slightly differ, nevertheless, the asphalt mix behaviour will always be the same. It is visible in Figure 7a that the stiffness modulus significantly decreases when the vehicle speed decreases. This decrease is especially rapid for low speeds, bellow 20 km/h. It can be concluded from Figure 7b that the stiffness modulus of asphalt concrete decreases up to 7 times when the temperature increases from 4 °C to 40 °C. It is also visible that temperatures affect the stiffness modulus more significantly than the vehicle speed. Such behavior is a consequence of the visco-elastic nature of asphalt mixtures, which is emphasized at higher temperatures and lower speeds.

The analysis includes only the effect of the asphalt mix stiffness modulus variability. However, it must be noted that the module of subbase and subgrade of the road are not constant during the year. The subbase and subgrade change their mechanical parameters as a result of moisture, frost and soil properties, but these effects are not considered in this work.

The pavement layer thickness affects stresses and strains induced by traffic loads significantly. Thicker structures make a stiffer fulcrum for load sensors in the WIM, while in thinner structures the support for the axle load sensor is less stiff. The consequences of the pavement thickness are deflections, which are larger for thinner pavements. For this analysis one pavement structure was considered, in order to make the analysis clearer and to expose the thermal and speed effects on vehicle weighing. The effect of pavement thickness were not considered. The thicknesses and mechanical parameters of particular layers in the pavement structure—assumed for analyses—are given in Table 1.

### 4.3. Pavement Deflections

**Figure 8:**
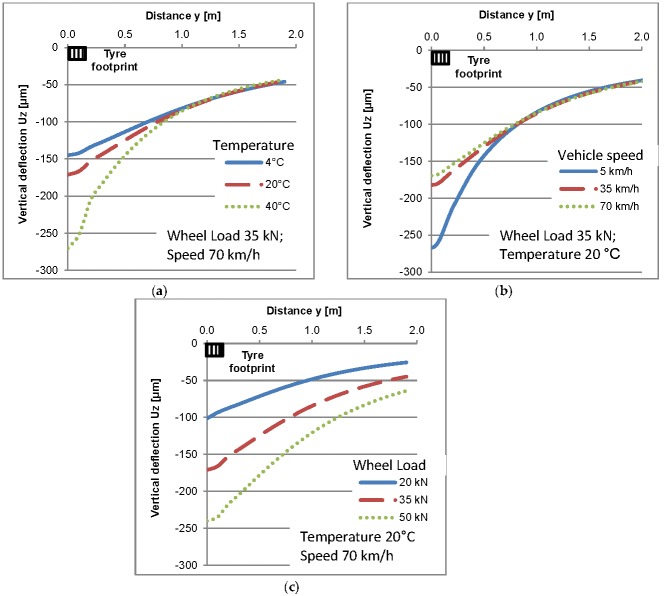
Pavement surface deflections under: (**a**) various temperatures and a constant wheel load and speed; (**b**) various speeds and a constant wheel load and temperature; (**c**) various wheel loads and a constant temperature and speed.

Deflections of the assumed pavement structure under constant wheel load and at three various temperatures are shown in Figure 8a. As it can be concluded, under the constant wheel load an increase of temperature causes an increase of deflections and changes in their shapes. Figure 8b shows vertical deflections under a constant wheel load and temperature but at different vehicle speeds from 5 km/h to 70 km/h. Figure 8c shows deflections under different wheel loads at a constant, moderate temperature of 20 °C and average vehicle speed of 70 km/h.

The scale of deflection changes under different temperatures and speeds is comparable to deflection changes under different wheel loads. For example, an increase of temperature from 20 °C to 40 °C or a decrease of vehicle speed from 70 km/h to 5 km/h causes a higher change of the vertical deflection of the pavement than a wheel load increase from 35 kN to 50 kN. What is important is that the deflection shape is different when either speed or temperature is variable (Figure 8a,b) in comparison to the deflection shape under various loads (Figure 8c). This indicates that the stress distribution inside the pavement structure changes when either temperature or speed changes. This means that stresses applied directly to the WIM sensor (see Figure 5) will be different under variable temperature and vehicle speed even if the wheel load and contact stress are constant.

### 4.4. Analysis of Vertical and Horizontal Stresses Acting on WIM Sensors 

**Figure 9:**
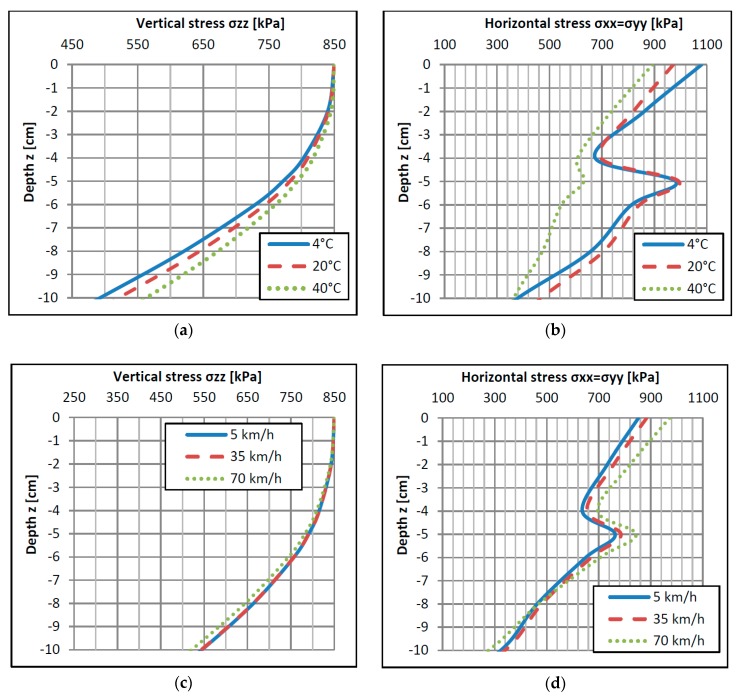
Distribution of normal stresses on the pavement structure depth under wheel load *p* = 35 kN, contact stress q = 850 kPa: (**a**,**b**) at a constant speed of 70 km/h and various temperatures; (**c**,**d**) at a constant temperature of 20 °C and various vehicle speeds.

As we mentioned before, the vehicle’s wheel load is the source of the contact stress (on the pavement/sensor top) and reaction stress (on the sensor fulcrum). Figure 9 presents distributions of vertical stresses (Figure 9a,c) and horizontal stresses (Figure 9b,d) under an applied load at different depths *z* under the road surface. Stress distributions presented in Figure 9 were calculated for wheel load *P* = 35 kN and a contact stress between the tyre and pavement *q* = 850 kPa. The distributions in Figure 9 are presented for different depths because in different techniques of WIM sensor installation, the depth of the sensor top and its fulcrum can be different. Figure 9 is universal to estimate the error of the WIM sensor for any technique of its installation, however the example described below is performed for most the popular quartz sensor.

In the case of sensors installed at the pavement surface level (e.g. bending plate, quartz), the vertical stress on the top of the pavement $\sigma_{zz}^{top}$ is equal to the contact stress *q*, regardless of the wheel load value *P* (compare Figure 9 and Figure 5). The vertical stress at the depth of the sensor fulcrum $\sigma_{zz}^{fulcrum}$ is lower than vertical stress at the level of sensor top (Figure 9a,c). The characteristics in Figure 9 prove that the temperature change and the vehicle speed cause the axle load measurement error. The error source is a difference between stresses $\sigma_{zz}^{top}$ and $\sigma_{zz}^{fulcrum}$. Stress $\sigma_{zz}^{fulcrum}$ depends on the temperature (Figure 9a) and speed (Figure 9c) while $\sigma_{zz}^{top}$ is constant, except for polymer sensors mounted under the road surface for which $\sigma_{zz}^{top}$ also depends on the temperature and speed.

Another aspect is constituted by horizontal stresses affecting the axle load sensor (see Figure 9b,d). The values of horizontal stresses are comparable to vertical stresses but they are more sensitive to temperature and speed variations than vertical stresses. Horizontal stresses can impact measurements of the wheel load in the WIM sensor, which was confirmed in field observations [5,27]. The mechanism of an error occurring is analogous to the error caused by vertical stresses. Changes of measurement conditions (temperature and speed) in relation to conditions during the WIM system calibration will cause a change of the horizontal stress distribution. In the case of horizontal stresses, both stress values—i.e., on the sensor fulcrum and on the whole height of the sensor—can have an impact on the wheel load measurement. The effect of horizontal stresses is especially visible for polymer sensors mounted under the road surface. The producer of the quartz sensor declares that the shape of the aluminum sensor extrusion (compare Figure 3) allows the influence of horizontal stresses on the axle load measurement to be minimized.

The analysis of the vertical and horizontal stresses acting on the WIM sensor with different temperatures and vehicle speeds can be used to improve the accuracy of wheel load measurements. For this purpose, the mechanical properties of asphalt layers in a specific location of the WIM station should be obtained. The FWD test device or laboratory tests of specimens of asphalt mixtures cored out from the pavement can be used for this purpose. The mechanistic analysis of the pavement structure allows us to determine the ratio *p* between stresses acting on the sensor top and fulcrum, as a function of temperature and vehicle speed. The relative change of ratio *p* caused by change in temperature and vehicle speed in comparison to the conditions during the calibration of the WIM system is a new calibration factor for the measurement of vehicle wheel loads.

### 4.5. Case Study 

**Figure 10:**
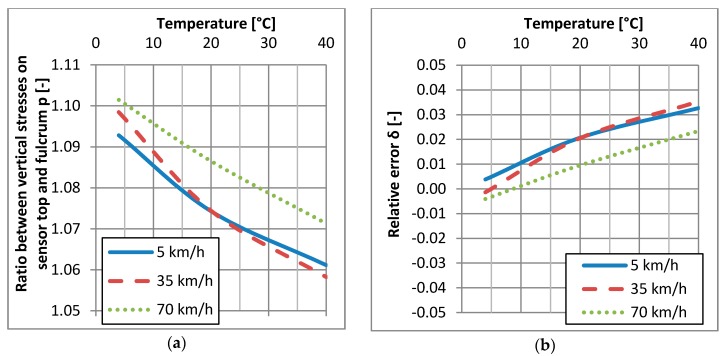
Example of theoretical analysis of relative error occurrence on the WIM station with quartz sensors (**a**) ratio *p* between the vertical stresses *σ_zz_* on the surface level and on the level of the sensor fulcrum (**b**) relative error *δ* caused by the change of pavement temperature and vehicle speed in relation to conditions during calibration of the WIM station (T = 20 °C and v = 70 km/h).

**Figure 11:**
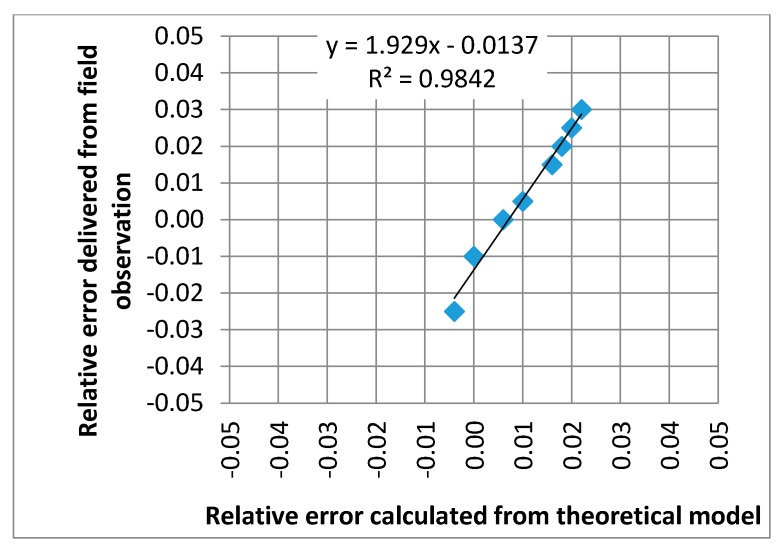
Comparison of relative errors for the quartz sensors delivered from field observations and from the theoretical model for the range of temperatures from 5 °C to 40 °C.

Figure 10 illustrates an example of the calculation of measurement error for quartz sensors from the elastic-half space model. It was assumed that error is caused only by the effects of differences between vertical stress on the surface level (*z* = 0 cm) and on the level of the sensor fulcrum (*z* = 5 cm), which is expressed by ratio $p=\frac{\sigma_{zz}^{top}}{\sigma_{zz}^{fulcrum}}$. The ratio *p* is a function of speed and temperature as given in Figure 10a. Let’s assume the following conditions of the calibration of the WIM system: vehicle speed 70 km/h and pavement temperature 10 °C what yields the relative error *δ* = 0 in Figure 10b. For calibration conditions the ratio *p* equals 1.097. When the pavement temperature and the vehicle speed change, the ratio *p* will also change and will consequently cause measurement error. The relative change of ratio *p* corresponds to the relative error which is given in Figure 10b. The error provided from theoretical analysis, given in Figure 10b, corresponds to a similar level of the relative error of the wheel load measurement, given in Figure 4 for quartz sensors. The relationship between the relative error calculated from the model and obtained from the real WIM site is presented in Figure 11. It should be noted that the error delivered from theoretical analysis includes only one factor: the change of vertical stresses in the pavement structure caused by the stiffness modulus *E* change in the asphalt mixes. This can explain the slightly higher values of relative errors delivered from field observation. The real conditions of the pavement structure are much more complex, which additionally increases measurement error.

## 5. Summary and Conclusions

The studies presented in the paper proved that the weighing accuracy in Weigh-in-Motion systems depends on the pavement/sensor complex behavior under dynamic loads of the vehicle axle. In the case of WIM systems, the pavement constitutes a part of the measuring system, and pavement properties affected the weighing result accuracy. Thus, the accuracy of the WIM system should not be described exclusively by the accuracy of the sensor itself but by the pavement/sensor complex properties. The analysis presented in the paper concerns only flexible (asphalt) pavements. The presented analysis leads to the conclusions given below.

1. Field observations indicated the evident influence of temperature and vehicle speed on the relative error of weighing results. In the case of quartz and bending plate sensors, a temperature change within the range −10 °C to +30 °C produced a weighing error change of approximately 7%. Weighing results for the same vehicle traveling at speeds of 55 km/h and 85 km/h differed by 6% for polymer sensors, by 4% for quartz sensors and by 1% for bending plate sensors.
2. Polymer sensors had the worst properties and should not be used for direct enforcement purposes. For this type of sensor, a temperature change caused a change in the weighing error of approximately 50%.
3. The source of the error observed in the field was identified with the use of pavement mechanics theory. In general, differences in the distribution of vertical and horizontal stresses in the pavement structure are the main reasons for the weight measurement error.
4. Differences in stress distributions result from variability of the modulus of stiffness *E* of asphalt layers whose value significantly depends on temperature and vehicle speed.
5. The shape and values of the pavement surface deflection results directly from stress distributions, and illustrate well the effect of variations of temperature and speed. Analyses of the shape of the pavement surface deflection and its maximum value indicate that stress distributions inside the pavement structure significantly change when temperature or vehicle speed varies.
6. The ratio between vertical stresses applied to the sensor top and its fulcrum has the greatest effect on the measurement error in the case of quartz and bending plate sensors. The analysis of the theoretical model provided similar results in relative error as the field observations and it equals up to 3% for the range of temperature from 4 °C to 40 °C and the range of vehicle speed from 5 km/h to 70 km/h. The horizontal stresses are more sensitive to temperature and speed variations, and they have an additional effect on polymer sensors. The relative error for polymer sensors is several times higher in comparison to quartz and bending plate sensors.
7. The analysis proves that thermal and speed effects cannot be neglected during the WIM systems calibration process. This paper describes the source of the measuring error observed in the field, which can contribute to the improvement of the Weigh-in-Motion system accuracy.

The described phenomena concern all kinds of axle load sensors embedded in the pavement: polymer, quartz and bending plate. Even in the case of the quartz load sensors, which are considered to be the best ones, the output signal depends on the pavement temperature and vehicle speed. Therefore, in the authors’ opinion: (1) It is necessary to reconsider views about the WIM system calibration method proposed in COST323 [41], (2) Achieving the high and constant accuracy of the WIM systems requires the implementation of temperature and/or speed correction algorithms of weighing results or an implementation of the auto-calibration method of the system.

## Figures and Tables

**Table 1:** Thicknesses and mechanical parameters of particular pavement layers assumed for analyses.

Layers of the Pavement Structure			Vehicle Speed (Km/h)	Stiffness Modulus E_i_ (MPa) at Temperatures			Poisson Ratio v_i_ (-) at Temperature		
No.	Thickness h_i_ (CM)	Name and Material		4 °C	20 °C	40 °C	4 °C	20 °C	40 °C
1	4.0	Wearing course (SMA 45/80-55)	5	10,800	3500	800	0.25	0.35	0.40
			35	12,600	4400	1100			
			70	14,900	6300	1600			
2	6.0	Binding course (AC 16 W 35/50)	5	21,400	8000	900	0.25	0.35	0.40
			35	24,300	9700	1400			
			70	26,300	13,600	2300			
3	10.0	Asphalt base (AC 22 P 35/50)	5	23,500	12,500	1400	0.25	0.35	0.40
			35	25,100	15,800	2700			
			70	28,100	18,000	3800			
4	20.0	Granular subbase (crushed stone)	All ranges	400			0.3		
5	∞	Subgrade	All ranges	120			0.35		
